# Autonomous motivation, goal-facilitating behaviours, and dietary goal progress in individuals transitioning to a veg*n diet: A longitudinal study

**DOI:** 10.3389/fpsyg.2022.1019714

**Published:** 2022-12-21

**Authors:** Marta Kolbuszewska, Jo Anderson, Marina Milyavskaya

**Affiliations:** ^1^Department of Psychology, University of British Columbia, Vancouver, BC, Canada; ^2^Department of Psychology, Carleton University, Ottawa, ON, Canada; ^3^Faunalytics, Olympia, WA, United States

**Keywords:** vegan, vegetarian, self-determination, autonomy, behaviour change

## Abstract

Previous studies have consistently shown that autonomous motivation – pursuing goals because one *wants to*, rather than *has to* – is associated with greater behaviour maintenance in the context of healthy eating, exercise, and diet maintenance. The present study used a 7-wave longitudinal design to examine how autonomous motivation is related to dietary goal progress in individuals (*N* = 222) transitioning to a veg*n (i.e., vegetarian or vegan) diet. We hypothesized that when people reported more autonomous motivation (compared to their own average) they would be more successful in reaching their dietary goals. We also explored the role of goal-facilitating behaviours in this process. We found no directional effects of relative autonomous motivation on goal progress or goal-facilitating behaviours, although the concurrent relations were significant. There were also no within-person effects of behaviours on progress. These findings shed light onto the relationship between autonomous motivation, behaviours, and goal progress both at the same time and over time, and highlight the importance of examining within-person fluctuations over time.

## Introduction

Plant-based diets (whether in the form of reduced meat consumption or complete elimination of animal products) have received increased media coverage and popular interest in recent years (Phua et al., 2020; Van Loo et al., 2020). Campaigns to reduce meat consumption like Veganuary have seen growing success; in 2022, 629,000 people participated in Veganuary, up from 3,000 in 2014 (Veganuary, 2022), and meat alternatives are now mainstream, available at fast-food chains and grocery stores (Van Loo et al., 2020). Indeed, reducing meat consumption provides significant opportunities to mitigate climate change and reduce greenhouse gas emissions (Shukla et al., 2019), protect animal welfare (Westhoek et al., 2014), and reduce the burden of chronic illness linked to cardiovascular disease (Kahleova et al., 2018; Siapco and Sabaté, 2019), colorectal cancers (Bouvard et al., 2015), and type 2 diabetes (Kahleova et al., 2011). Nevertheless, global meat consumption is increasing (Godfray et al., 2018) and despite considerable interest in vegetarian or vegan (henceforth referred to as veg*n) diets, only a small proportion of individuals who try a veg*n diet stick with it long-term (Faunalytics, 2016). In the United States, there are more than five times as many former veg*ns (i.e., people who tried a veg*n diet and then abandoned it) as current veg*ns; that is, over 80% of vegetarians/vegans abandon their diet (Faunalytics, 2016). Given the low success rate, what can help people stick to a veg*n diet?

### Autonomous motivation

A large body of research has explored the environmental and psychological factors that influence individuals’ uptake and maintenance of health behaviours (Sallis et al., 2015). Motivation is a psychological factor that is especially relevant to promoting goal attainment (Sheldon et al., 2004). Although past research has examined different motives or reasons for transitioning to a veg*n diet (Fox and Ward, 2008; Faunalytics, 2014; Grassian, 2020; North et al., 2021; Ghaffari et al., 2022), it has not considered the *quality* of motivation. Self-Determination Theory (Deci and Ryan, 2000) suggests that motivation exists on a continuum from autonomous (because one *wants to*) to controlled (because one *has to;* Ryan and Connell, 1989). Autonomous motivation entails enacting a behaviour for the enjoyment or challenge inherent to that action, because it is integral to their identity (e.g., a person avoids meat because it aligns with their beliefs), or because they believe their goal to be important (e.g., a person reduces milk consumption because they do not support the dairy industry, even though they really like cheese). Controlled motivation, on the other hand, is characterized by external pressures and demands. Individuals might pursue a goal because of feelings of guilt, shame, or social pressure (e.g., a vegetarian gives up eggs and dairy because they fear the negative reactions of their vegan peers if they continue eating animal products), or in order to meet external incentives or to satisfy an external demand (e.g., a person eats vegetarian because they will win a gift certificate if they do so).

Past studies have found that more autonomous motivation is associated with greater goal progress (Sheldon and Elliot, 1999; Koestner et al., 2002; Sheldon et al., 2004; Werner et al., 2016), and behaviour maintenance (of multiple behaviours; Ryan and Connell, 1989; Silva et al., 2011; Hagger et al., 2014; Nurmi et al., 2016; Vancampfort et al., 2016; Voi and Sainsbury, 2019), compared to controlled motivation. Although some of this research has examined the role of autonomous and controlled motivation in diet maintenance, it has predominantly focused on dieting for weight loss, which entails caloric restriction (Wadden et al., 1994; Wing et al., 2006) and implicates physiological processes related to metabolic adaptation that may interfere with weight loss maintenance (Ohsiek and Williams, 2011; Anastasiou et al., 2015). Transitioning to, and maintaining, a veg*n diet can thus differ from pursuing diets for weight control. Additionally, autonomous as opposed to controlled motivation is associated with using more adaptive strategies (Koestner et al., 2008), experiencing fewer temptations (Milyavskaya et al., 2015) and reporting greater subject ease (Werner et al., 2016) during general goal pursuit. We thus expect that relatively more autonomous motivation will similarly relate to more successful transition to a veg*n diet, including engagement in more goal-facilitating behaviours.

### Goal-facilitating behaviours

What are some ways that people can stick to their goal of eating fewer animal products? Engaging in certain activities, like planning meals in advance, or avoiding situations with tempting dietary options, can help people succeed at meeting their goals (Williamson and Wilkowski, 2020). For veg*ns, some activities may be more influential than others at helping people reach their dietary goals. Faunalytics (2014) and Grassian (2020) found that veg*ns and former veg*ns list six general factors that influence veg*n diet maintenance, including cravings, convenience, motivation, cost, health concerns, and social support.

#### Strategies for dealing with cravings

One way to promote goal-consistent choices is by using strategies to actively manage oneself and one’s environment and avoid temptations that may hinder goal progress (Duckworth et al., 2014; Duckworth et al., 2016). Prior research on self-control strategies finds that they are generally effective in preventing indulgence in the moment, and that using more strategies is more effective (Milyavskaya et al., 2020). In the context of transitioning to a veg*n diet, individuals may use strategies when faced with tempting situations (e.g., planning a strategy to use if a craving occurs) in order to help achieve their goals.

#### Convenience

Goal-consistent behaviours that increase convenience may also play an important role in supporting individuals’ transition and maintenance of veg*n diets. For example, *situation selection*, choosing situations that help one stick to their goals and avoiding situations where self-control will be needed, helps people stick to their various goals more broadly (Duckworth et al., 2016). Similarly, choosing in advance generally encourages more self-controlled decisions (Laibson, 1997). Consequently, behaviours that increase convenience (e.g., going to a vegetarian restaurant instead of a steakhouse) may be particularly relevant to those following a veg*n diet.

#### Information seeking

Veg*ns cite various reasons for eliminating animal products from their diet, with animal welfare, environmental, and health concerns often topping the list (Dyett et al., 2013; Kerschke-Risch, 2015; Radnitz et al., 2015; Izmirli and Phillips, 2011, see Janssen et al., 2016). However, some reasons are more effective than others at influencing long-term behaviour change; for example, many veg*ns cite learning about the harms of livestock agriculture as an important factor in transitioning to a veg*n diet (Faunalytics, 2014). We thus examined whether learning about the benefits of a veg*n diet (or the potential detriments of a non-veg*n diet) promotes dietary change.

#### Health

Another set of behaviours that are particularly relevant to the veg*n context are health-related behaviours (e.g., getting blood work done to check one’s iron or B12 levels). Monitoring nutritional needs is especially important for maintaining health and may thereby help people stick to their diet (Herrmann and Geisel, 2002). Regular monitoring of serum B12 and iron levels has been recommended for children and pregnant/lactating women (Lemale et al., 2019), as well as adults (Herrmann and Geisel, 2002; Pawlak et al., 2014) following a plant-based diet, and current veg*ns are much more likely to have had these values checked than those who abandon a veg*n diet (Faunalytics, 2014). Additionally, nutritional concerns and deficits are cited as one of the top reasons for abandoning a veg*n diet (Faunalytics, 2014). As such, proactively monitoring health and nutrition may help individuals successfully transition to a veg*n diet.

#### Cost

For individuals pursuing a healthy diet, food choice is influenced by personal economic conditions: Eating a healthy, and environmentally friendly, diet often incurs financial costs (Cade et al., 1999) while financial constraints lead to less healthy eating (Drewnowski and Darmon, 2005; Barosh et al., 2014). Examining the affordability of health food items, Barosh et al. (2014) found that a low-income household would need to spend 40 to 48% of their weekly income to afford a healthy food basket, whereas a high-income household would only need to spend between 8 and 9% of their salary to afford the same food. Although veg*n and reduced-meat diets do not necessarily need to be expensive (Wilson et al., 2013), many individuals transitioning to a veg*n diet find cost to be a factor when planning their diet (Faunalytics, 2014; Van Den Berg et al., 2022). Strategies to reduce costs may therefore play a role in successfully transitioning to and maintaining a veg*n diet.

#### Social

Social support from various sources (e.g., family, healthcare professionals, friends) can positively influence goal attainment, including health outcomes (Jakubiak and Feeney, 2016). Social support is implicated in healthier food choices (Kubik et al., 2005; Stanton et al., 2007) and adherence to dietary changes (Sorensen et al., 1998; Wilson et al., 2013). Just as diet-related social support helps individuals pursue healthy eating behaviours, social support plays an important role in maintaining a veg*n diet (Jabs et al., 1998; Hielkema and Lund, 2021), with veg*ns often seeking out veg*n social groups (Chuter, 2018; Séré de Lanauze and Sirieix, 2022) and romantic partners (Twine, 2014). Similarly, a lack of social support can present a barrier to individuals maintaining a veg*n diet (Hodson and Earle, 2018; Markowski and Roxburgh, 2019).

### Fluctuations over time and the present research

There is evidence that goal intentions (Conner et al., 2000; Conroy et al., 2011) and behaviours implicated in goal pursuit (Inauen et al., 2016) naturally fluctuate over time. That is, people’s intentions (e.g., to exercise) and behaviours (e.g., snacking) vary on the short- to medium- term (i.e., on the weekly and monthly scale; Conroy et al., 2011; Reuter et al., 2009; Scholz et al., 2008). Previous research found that the greatest reported drop-off in diet adherence was within the first few months of a veg*n diet (34% within 3 months, another 19% within the first year; Asher et al., 2014); we thus chose one-month follow-ups, for 6 months, to try and strike a reasonable balance between frequency in those early months and not overburdening participants. Our research addresses a critical gap in the literature by addressing how goal progress may vary for a single individual over time, examining prospective effects of within-person deviations from trait levels. More concretely, we examine whether relative autonomous motivation will lead to engaging in more behaviours that facilitate goal pursuit, and whether this will lead to greater dietary goal progress among individuals transitioning to a veg*n diet - that is, whether more relative autonomous motivation in a given month compared to *your own average* will lead to greater goal progress).

### The current study

In the present research, we examined whether feeling greater relative autonomous motivation and engaging in more behaviours that facilitate goal pursuit predict dietary goal progress among individuals transitioning to a veg*n diet. Specifically, we were interested in assessing whether goal-facilitating behaviours mediate the relationship between motivation and progress. We hypothesized that: (A) When individuals have higher relative autonomous motivation than usual, they will experience a subsequent increase in dietary goal progress. (B) When individuals engage in more behaviours that facilitate goal progress than usual, they will experience a subsequent increase in dietary goal progress. (C) Individuals who have higher relative autonomous motivation than usual will engage in more behaviours that facilitate goal progress than usual; this will in turn lead to an increase in dietary goal progress. (D) There will be an indirect effect of relative autonomous motivation on goal progress *via* behaviours that facilitate goal progress at the within-person level.^1^

## Materials and methods

### Participants and procedure

Participants were 222 individuals transitioning to a vegetarian or vegan diet (67.6% women, M_age_ = 31.4, 40.5% had attempted a veg*n diet before, average time since beginning the current veg*n diet = 3.52 weeks) recruited from a variety of North American online sources (e.g., Facebook groups for health, plant-based recipes). Participants were surveyed once a month for 6 months, starting with a baseline survey at sign-up. At each follow-up, participants were emailed to ask if they were still pursuing the diet. If they were not pursuing their diet anymore, they were directed to a separate survey regarding diet abandonment. If they were still pursuing the diet, they filled out a longer survey.^2^ Therefore, participants completed up to seven surveys in total. All survey questionnaires are available at https://osf.io/bhksj/. Sample size was determined by *a-priori* power analyses, with a sample of 200 participants required to detect a small-to-medium effect (*f*^2^ = 0.08) with 90% power at α = 0.05 level of significance (although we did initially plan to recruit 400 participants to account for drop-out; see https://osf.io/z5vef/ for the original recruitment plan). Our final sample consisted of 222 participants. Exclusion criteria were pre-registered, however due to an influx of scammers during initial stages of data collection, exclusion criteria were revised prior to analysis to remove fraudulent responses. Full details of the revised exclusion criteria can be found online at https://osf.io/bhksj/. The data were collected as part of a collaboration with Faunalytics (a non-profit organization), which has published two reports stemming from the full dataset (Anderson et al., 2021; Anderson and Milyavskaya, 2021).

### Measures

#### Relative autonomous motivation

A 12-item adapted version of the Treatment Self-Regulation Questionnaire (TSRQ; Ryan and Connell, 1989) was used to measure relative autonomous motivation for maintaining a veg*n diet. This scale consists of 12 items that ask participants to rate six statements representing autonomous motivation (e.g., “Because I personally believe it is the best thing to do”) and six statements representing controlled motivation (e.g., “Because I would feel guilty or ashamed of myself if I did not follow this diet”) on a 5-point Likert Scale ranging from 1 = strongly disagree, to 5 = strongly agree. For the purposes of the present study, relative autonomous motivation was computed as a composite score of the TSRQ, by subtracting the score for controlled motivation from autonomous motivation. Reliability for both the controlled motivation (*α* = 0.75–0.88) and autonomous motivation (*α* = 0.86–0.92) subscales was high.

#### Behaviours that facilitate goal progress

To assess behaviours that facilitate goal progress, participants were provided a list of 44 items, divided into six subsections: social (7 items; e.g., “Tried to meet new people with diets similar to yours”), convenience (8 items; e.g., “Switched to a restaurant, dining hall, etc., with better options for your diet”), cost (4 items; e.g., “Looked for cheaper restaurants”), health (5 items; e.g., “Taken vitamins or nutritional supplements”), information (9 items; e.g., “Learned more about the environmental impact of eating meat”), and cravings (11 items, e.g., “Planned a strategy for dealing with temptation if it occurs”). The total of 44 items included one open-ended item in each subsection. All variables were binary, scored 1 = “Yes,” 0 = “No,” that participants engaged in over the past month. The open-ended item was a string entry and was recoded as “yes” if answered (and answer did not duplicate prior answers), “no” if unanswered. A total score for behaviours was obtained by summing total items checked for each month.^3^

#### Dietary goal progress

We assessed dietary goal progress using two measures: subjective dietary goal progress and objective dietary goal progress.^4^

#### Subjective dietary goal progress

Dietary goal progress was assessed using a measure of perceived (subjective) goal progress. Participants were asked to rate how much progress they had made toward their dietary goal using a scale from 0% (not at all successful) to 100% (completely successful).

#### Objective dietary goal progress

Objective goal progress was calculated as a difference between the *goal diet* at the initial time point and the *actual diet* at the given time point, both assessed using a Food Frequency Questionnaire, a type of structured recall instrument often used in epidemiology research (e.g., Fred Hutch Cancer Center, 2022; National Cancer Institute, 2022) and more recently in psychology (e.g., Asher and Peters, 2020; Sparkman et al., 2021). At baseline, before all participants had started working towards their goal, objective goal progress was calculated as a difference between their goal diet and their actual diet at the same time point. The instructions for reporting *dietary goals* were as follows: “Once you achieve your new goal, how often do you expect to eat each of the following foods (including in other dishes or baked goods)?” Participants were provided with 5 food groups (dairy, poultry, fish/seafood, red meat, eggs) and asked to rate each item on a 5-point scale ranging from 1 = daily to 5 = not at all.

The instructions for reporting *actual diet* were as follows: “In the past month, how often have you eaten each of the following foods (including in other dishes or baked goods)?” Participants were provided with 5 food groups (dairy, poultry, fish/seafood, red meat, eggs) and asked to rate each item on a 5-point scale ranging from 1 = daily to 5 = not at all. A composite score was calculated by taking a difference between goal and actual diet for each food category and then averaging the scores for each participant. All participants who ‘exceeded’ their dietary goals (i.e., those that ate *less* meat, fish, etc. than they cited as their goal) were calculated as having no difference between their goal and actual diet progress. Therefore, the range of possible scores is 0 to 4, with 4 representing the most progress.

### Analyses

**Figure 1:**
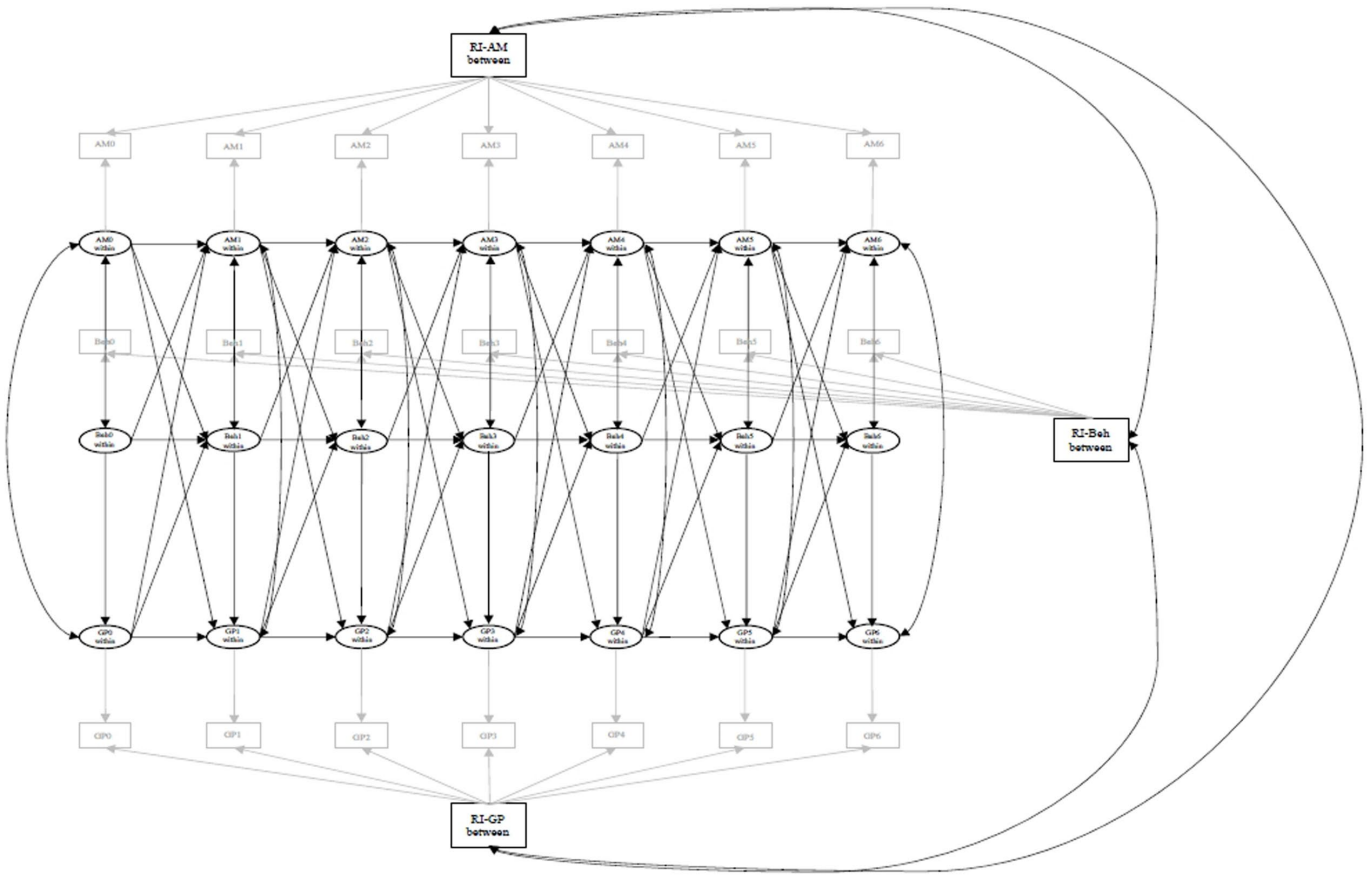
Random intercept cross-lagged panel model used to test hypotheses A to D.

All hypotheses as well as the analytical plan were pre-registered after data was collected but before data cleaning or analysis took place.^5^ Analyses were conducted in R version 4.0.2 (R Core Team, 2020) Hypotheses A, B, C, and D were tested using the RI-CLPM model specified in Figure 1. Since both behaviours and progress were assessed with respect to the past month, we tested effects of behaviours on progress reported at the same time point (rather than cross-lagged), since it would not have made sense for behaviours in 1 month to predict progress in the following month (for example, behaviours in January should predict progress in January, not in February). Three random intercepts account for stable trait-like differences between persons in relative autonomous motivation, behaviours, and goal progress and separate out between-person variance, allowing the lagged relationships to account for within-person variance. These random intercepts, with all factor loadings constrained to 1, are represented by RI-AM, RI-Beh, and RI-GP in Figure 1. Observed measures for goal progress, behaviours, and relative autonomous motivation were regressed onto their respective latent, within-person centered variables. As such, cross-lags and autoregressive lags between latent variables indicated how changes in one construct (from an individual’s average) influenced changes in other constructs (from an individual’s average). Intervals between waves had the same length, and so cross-wave equality constraints were placed on autoregressive and cross-lagged effects (Hamaker et al., 2015; Orth et al., 2020). Hypothesis D was tested by requesting indirect effects of motivation on progress *via* behaviour with bootstrapped confidence intervals.

As per the preregistration, we tested correlations between objective and subjective progress over time in order to determine whether an indicator variable would be created to measure goal progress. Shapiro–Wilk’s test was conducted to assess normality of data, and Spearman’s rho was used to calculate correlations due to the skewed nature of variable distributions. Correlations between objective and subjective progress ranged between *r* = 0.51 and *r* = 0.64 (see online supplement for correlations at each time point), under our pre-registered cut-off of *r* = 0.7. Consequently, we fit two models to the data, one with each measure of progress.

In both models, longitudinal associations between relative autonomous motivation, goal-consistent behaviours, and goal progress (either objective or subjective) across seven time points were modeled using lavaan in R.^6^ Although the original preregistration stated that all autoregressive, cross-lagged, and correlational paths would be constrained to be equal, at baseline (T0) many participants had not started working towards their goal and so instead we allowed the paths from goal progress at baseline to vary freely and constrained the paths across remaining time points. Model results with all paths constrained according to the original preregistration can be found in the online supplement.

In all analyses, full information maximum likelihood (FIML) estimates were used to deal with missing data (Enders and Bandalos, 2001). We assessed model fit using Comparative Fit Index (CFI), Root Mean Square Error of Approximation (RMSEA), Standardized Root Mean Square Residual (SRMR), and Chi square statistics. Because significance levels of the Chi-square statistic depend on sample size, model fit was evaluated using CFI, RMSEA, and SRMR. CFI values greater than 0.90 were considered to indicate acceptable fit, greater than 0.95 good fit. RMSEA values less than 0.08 indicated good fit, and SRMR values less than 0.06 indicated good fit (Hu and Bentler, 1999). For all analyses, standardized results are reported in text; unstandardized results can be found in the online supplement.^7^

## Results

### Preliminary analyses

Descriptive statistics of the variables of interests are presented in Table 1, and correlations are presented in Table 2.

**Table 1:** Means, standard deviations, and ICC of variables of interest at times T0-T6.

	Time							
	T0	T1	T2	T3	T4	T5	T6	
**Variable (range)**	**M (SD)**	**M (SD)**	**M (SD)**	**M (SD)**	**M (SD)**	**M (SD)**	**M (SD)**	**ICC**
AM [−5–5]	1.95 (0.92)	1.81 (0.95)	1.80 (0.95)	1.69 (0.92)	1.52 (0.93)	1.53 (1.09)	1.57 (1.04)	0.57
GFB [0–44]	16.45 (6.99)	16.48 (6.86)	16.02 (7.25)	16.14 (7.68)	16.17 (8.18)	15.97 (8.96)	16.77 (9.05)	0.74
GPO [0–4]	2.70 (0.76)	3.61 (0.49)	3.68 (0.42)	3.70 (0.38)	3.73 (0.35)	3.76 (0.26)	3.78 (0.31)	0.30
GPS [0–100]	76.01 (21.19)	76.49 (2.73)	80.59 (17.90)	83.24 (17.23)	85.29 (15.70)	86.13 (15.60)	88.63 (14.18)	0.55

AM = Relative Autonomous Motivation; GFB = Behaviours; GPO = Objective Goal Progress; GPS = Subjective Goal Progress; ICC = Intraclass correlation.

**Table 2:** Correlation matrix of all variables at times T0-T6.

Variable		Objective Goal Progress							Subjective Goal Progress							Autonomous Motivation							Goal-Facilitating Behaviours						
	**Time**	**0**	**1**	**2**	**3**	**4**	**5**	**6**	**0**	**1**	**2**	**3**	**4**	**5**	**6**	**0**	**1**	**2**	**3**	**4**	**5**	**6**	**0**	**1**	**2**	**3**	**4**	**5**	**6**
Objective Goal Progress	0	1																											
	1	0.19*	1																										
	2	0.21*	0.76***	1																									
	3	0.20*	0.74***	0.68***	1																								
	4	0.20*	0.77***	0.72***	0.83***	1																							
	5	0.04	0.51***	0.51***	0.61***	0.68***	1																						
	6	0.14	0.60***	0.59***	0.66***	0.63***	0.63***	1																					
Subjective Goal Progress	0	−0.12	0.36***	0.28**	0.29**	0.21*	0.29**	0.24*	1																				
	1	−0.08	0.50***	0.38***	0.34***	0.33***	0.35***	0.31**	0.56***	1																			
	2	−0.07	0.44***	0.52***	0.41***	0.35***	0.26*	0.25*	0.51***	0.67***	1																		
	3	−0.01	0.39***	0.43***	0.53***	0.48***	0.49***	0.32**	0.44***	0.50***	0.56***	1																	
	4	0.00	0.33***	0.34***	0.44***	0.56***	0.47***	0.23*	0.41***	0.48***	0.64***	0.71***	1																
	5	−0.11	0.38***	0.27**	0.47***	0.42***	0.53***	0.35***	0.43***	0.50***	0.57***	0.66***	0.71***	1															
	6	−0.03	0.31**	0.37***	0.38***	0.37***	0.34***	0.45***	0.33***	0.38***	0.42***	0.44***	0.47***	0.49***	1														
Autonomous Motivation	0	−0.08	0.16	0.14	0.10	0.14	−0.04	0.07	0.16*	0.08	0.07	0.09	0.14	0.21*	0.18	1													
	1	−0.03	0.17*	0.08	0.07	0.12	−0.05	−0.01	0.06	0.08	0.12	0.03	0.19	0.24*	0.05	0.52***	1												
	2	0.00	−0.04	0.03	−0.12	−0.08	−0.29**	−0.14	−0.07	0.02	0.09	−0.04	0.04	0.14	0.03	0.39***	0.50***	1											
	3	0.05	0.16	0.07	0.14	0.18	−0.08	0.01	0.04	0.11	0.15	0.17	0.19	0.15	0.01	0.45***	0.60***	0.56***	1										
	4	−0.12	0.04	0.01	0.03	0.11	−0.10	−0.10	0.04	0.04	0.10	0.18	0.22	0.15	−0.01	0.52***	0.58***	0.47***	0.67***	1									
	5	−0.18	−0.08	−0.19	−0.14	−0.04	−0.15	−0.22*	−0.09	−0.07	0.11	0.00	0.14	0.17	−0.04	0.46***	0.60***	0.60***	0.65***	0.73***	1								
	6	−0.16	−0.03	0.03	−0.03	0.05	−0.19	−0.09	−0.06	−0.02	0.18	0.11	0.18	0.18	0.11	0.40***	0.61***	0.56***	0.64***	0.74***	0.76***	1							
Goal-Facilitating Behaviours	0	−0.03	0.21*	0.28**	0.21*	0.12	0.23*	0.18	0.07	0.07	0.05	0.04	−0.06	−0.04	0.05	0.02	−0.13	−0.09	−0.14	−0.31**	−0.36***	−0.30**	1						
	1	−0.03	0.16	0.24*	0.13	0.13	0.22*	0.26*	0.19*	0.11	0.08	0.04	0.05	0.06	0.06	0.09	−0.08	−0.07	−0.11	−0.27*	−0.38***	−0.21*	0.69***	1					
	2	0.01	0.17	0.21*	0.15	0.08	0.20	0.22	0.03	−0.01	0.05	−0.05	−0.07	0.02	−0.02	−0.06	−0.15	−0.03	−0.08	−0.38***	−0.31**	−0.21	0.70***	0.78***	1				
	3	0.05	0.13	0.14	0.06	0.06	0.10	0.23*	0.03	0.06	−0.04	−0.08	−0.04	−0.05	−0.02	−0.03	−0.14	−0.05	−0.13	−0.34***	−0.32**	−0.24*	0.60***	0.75***	0.78***	1			
	4	0.06	0.14	0.19	0.10	0.08	0.09	0.19	0.13	0.08	−0.02	−0.09	0.00	−0.06	−0.01	−0.02	−0.25**	−0.20	−0.21*	−0.32***	−0.41***	−0.36***	0.62***	0.81***	0.83***	0.84***	1		
	5	0.10	0.10	0.15	0.12	0.05	0.13	0.12	0.10	0.01	−0.06	−0.04	0.03	0.02	0.04	−0.07	−0.29**	−0.13	−0.26*	−0.34***	−0.38***	−0.30**	0.60***	0.79***	0.80***	0.83***	0.90***	1	
	6	0.22*	0.21*	0.21*	0.17	0.02	0.06	0.21*	0.08	0.05	−0.08	−0.05	−0.13	−0.02	0.05	−0.03	−0.27**	−0.12	−0.24*	−0.38***	−0.42***	−0.30**	0.53***	0.73***	0.74***	0.77***	0.81***	0.87***	1

All *p* values corrected for multiple testing using FDR (Benjamini-Hochburg procedure).

* Denotes *p* < 0.05 level of significance.

** Denotes *p* < 0.01 level of significance.

*** Denotes *p* < 0.001 level of significance.

### Objective progress

The RI-CLPM model examining within-person relative autonomous motivation, behaviours, and objective goal progress showed adequate model fit (χ^2^ (202) = 406.76, *p* < 0.001, CFI = 0.91, RMSEA = 0.07, 90% CI (0.06, 0.08), although the SRMR = 0.13 was poor). Autoregressive paths for within-person relative autonomous motivation and behaviours were significant and moderate (see Table 3 for standardized parameters and online supplement for unstandardized parameters). The relationship between goal progress at baseline (before participants had started working towards their goal) and time 1 was negative (*β =* −0.24, *p = 0*.023) – unsurprisingly, participants who had already made more progress at baseline made smaller additional gains at the first follow-up. Autoregressive effects were larger than other reported effects, suggesting that relative autonomous motivation and engagement in goal-consistent behaviours over the past month are best predicted by the same constructs at the previous time point. No cross-lagged effects were significant. Indirect effects of motivation on goal progress *via* behaviours were not significant (*β =* −0.000, *p = 0*.996, 95%CI [−0.002, 0.002]).^8^ At the between-person level, the random intercept factor of relative autonomous motivation was uncorrelated with behaviours and goal progress suggesting that individuals who reported greater motivation did not necessarily report more behaviours or goal progress. The random intercept factors of behaviours and goal progress were positively correlated (*β = 0*.23, *p = 0*.008), such that those individuals who reported engaging in more behaviours overall also made more overall progress,

**Table 3:** Standardized RI-CLPM parameter estimates for Objective Progress Model.

Autoregressive paths (constrained T1-T6)	*β*	*p*-value	Cross-lagged paths (constrained T1-T6)	*β*	*p*-value	Covariances (constrained T1-T6)	*β*	*p*-value
AM_-1_ → AM_t_ (a1)	0.161	0.002**	GFB_t-1_ → AM_t_ (c1)	−0.058	0.305	AM_0_ ↔ GFB_0_	0.006	0.948
GFB_t-1_ → GFB_t_ (a2)	0.356	< 0.001***	GPO_0_ → AM_1_	−0.083	0.389	AM ↔ GFB	−0.080	0.062
GPO_t-1_ → GPO_t_ (a3)	0.218	< 0.001***	GPO_t-1_ → AM_t_ (c2)	0.018	0.731	AM_0_ ↔ GPO_0_	−0.073	0.376
GPO_0_ → GPO_1_	−0.237	0.023*	AM_t-1_ → GFB_t_ (c3)	0.001	0.990	AM ↔ GPO	0.108	0.011**
			GPO_0_ → GFB_1_	−0.127	0.164			
			GPO_t-1_ → GFB_t_ (c4)	−0.055	0.269			
			AM_t-1_ → GPO_t_ (c5)	0.017	0.677			
			GFB_t_ → GPO_t_ (c6)	−0.036	0.406			

AM = Autonomous Motivation; GFB = Goal-Facilitating Behaviours; GPO = Objective Goal Progress.

* *p* < 0.05;

** *p* < 0.01;

*** *p* < 0.001.

### Subjective progress

The RI-CLPM model examining relative autonomous motivation, goal-consistent behaviours, and subjective goal progress showed adequate model fit (χ^2^ (202) = 349.99, *p* < 0.001, CFI = 0.927, RMSEA = 0.057, 90% CI (0.047, 0.067), although SRMR was poor at 0.11). Autoregressive paths were all significant (see Table 4 for standardized parameters and online supplement for unstandardized parameters). Standardized autoregressive paths for within-person relative autonomous motivation (*β = 0*.16, *p = 0*.004), behaviours (*β = 0*.38, *p* < 0.001), and subjective goal progress (*β_T0_ = 0*.23, *p = 0*.016; *β_T1-T6_ = 0*.25, *p* < 0.001) were significant and low to moderate in size. Autoregressive effects were larger than other reported effects, suggesting that relative autonomous motivation and engagement in goal-consistent behaviours over the past month are best predicted by the same construct at the previous time point.

**Table 4:** Standardized RI-CLPM parameter estimates for Subjective Progress Model.

Autoregressive paths (constrained T1-T6)	*β*	*p*-value	Cross-lagged paths (constrained T1-T6)	*β*	*p*-value	Covariances (constrained T1-T6)	*β*	*p*-value
AM_t-1_ → AM_t_ (a1)	0.155	0.004**	GFB_t-1_ → AM_t_ (c1)	−0.066	0.246	AM_0_ ↔ GFB_0_	−0.007	0.937
GFB_t-1_ → GFB_t_ (a2)	0.377	< 0.001***	GPS_0_ → AM_1_	0.028	0.759	AM ↔ GFB	−0.074	0.085
GPS_t-1_ → GPS_t_ (a3)	0.248	< 0.001***	GPS_t-1_ → AM_t_ (c2)	0.153	0.006**	AM_0_ ↔ GPS_0_	0.200	0.023*
GPS_0_ → GPS_1_	0.231	0.016*	AM_t-1_ → GFB_t_ (c3)	0.001	0.979	AM ↔ GPS	0.102	0.018*
			GPS_0_ → GFB_1_	0.076	0.407			
			GPS_t-1_ → GFB_t_ (c4)	−0.064	0.233			
			AM_t-1_ → GPS_t_ (c5)	0.018	0.666			
			GFB_t_ → GPS_t_ (c6)	0.081	0.067			

AM = Autonomous Motivation; GFB = Goal-Facilitating Behaviours; GPS = Subjective Goal Progress.

* *p* < 0.05;

** *p* < 0.01;

*** *p* < 0.001.

Effects for subjective goal progress on motivation were significant (*β = 0*.15, *p = 0*.006), suggesting a small positive relationship between subjective goal progress and motivation (i.e., when individuals reported that they were more successful in attaining their goal compared to their own average, they reported more relative autonomous motivation compared to their own average at the following time). Covariances between relative autonomous motivation and goal progress at the same time point were significant across waves (*β_T0_ = 0*.20, *p = 0*.023; *β_T1-T6_ = 0*.10, *p = 0*.018). At the between-person level, the random intercept factors of relative autonomous motivation, behaviours, and goal progress had no significant correlations, suggesting that individuals who reported greater motivation did not necessarily report more behaviours or goal progress. Indirect effects of motivation on goal progress *via* behaviours were not significant (*β = 0*.002, *p = 0*.979, 95%CI [−0.122, 0.125]).

## Discussion

The primary aim of this study was to test the reciprocal associations between relative autonomous motivation, goal-facilitating behaviours, and dietary goal progress in individuals transitioning to a veg*n diet. Most of our hypotheses were not supported. At the within person level, contrary to hypothesis A, when individuals had higher relative autonomous motivation than usual, they did not report a subsequent increase in dietary goal progress (although motivation and progress were again positively correlated at the follow-ups). Contrary to hypothesis B, when individuals engaged in more behaviours that facilitate goal progress than usual, they did not report greater subjective or objective dietary goal progress. Inconsistent with hypothesis C, individuals who had higher relative autonomous motivation than usual did not report engaging in more behaviours that facilitate goal progress than usual. Lastly, contrary to hypothesis D, there was no significant indirect effect of relative autonomous motivation on goal progress *via* behaviours that facilitate goal progress at the within-person level.

### Theoretical and practical implications

Our findings add to a growing literature on veg*n diet transition that examines the role of motivations and behaviour in increasing veg*n diet maintenance (Faunalytics, 2016; Rosenfeld and Tomiyama, 2019a,b; Grassian, 2020). Furthermore, our results offer some support for past research examining the relationship between goal-consistent behaviours, goal-pursuit, and motivation (Judge et al., 2005; Koestner et al., 2008; Milyavskaya et al., 2015). The present research also provides a novel examination of both objective and subjective veg*n goal progress. This is particularly important in the context of a veg*n diet given that people’s claimed identity (e.g., vegan/vegetarian) does not always align with what they eat (e.g., 17% of vegetarians report regularly eating fish and 51% of vegetarians report having eaten meat since becoming vegetarian; Rosenfeld and Tomiyama, 2019a,b). Specifically, our study used an objective indicator of goal progress that took into account individuals’ idiosyncratic, specific goals. By considering the difference between people’s goals and actual diets, we were able to assess how much progress individuals had made related to *their own* goals (i.e., if two individuals stopped eating meat and ate only eggs, but one individual had a goal of eliminating only meat while the other individual had a goal of eliminating meat and eggs, our measure of goal progress accounted for these idiosyncrasies).

### Autonomous motivation

Our findings are partially inconsistent with empirical and theoretical work that identifies autonomous motivation as an important predictor of goal progress (e.g., Koestner et al., 2008; see Ryan and Deci, 2017 for an overview). As can be expected, the two constructs were positively related at each time point. This means that participants who experienced greater relative autonomy also reported more goal progress, and also in months where autonomy was higher, participants reported greater progress. However, relative autonomous motivation did not predict greater progress in the following month. This may be due to autonomous motivation being relatively stable (57% of variability was between-person) and high; perhaps it is only when motivation becomes relatively less autonomous that subsequent goal pursuit is affected. It is also likely that the goal of transitioning to a veg*n diet differs from other goals (e.g., veg*ns face social stigma for their choices; Markowski and Roxburgh, 2019), so the processes linking motivation to progress may differ, or may be moderated by factors we did not examine.

We did, however, find that goal progress was positively related to subsequent reports of relative autonomous motivation. This aligns with research by Sheldon and Houser-Marko (2001), who found that greater attainment of personal goals was linked to more self-concordance (i.e., within-person relative autonomous motivation) the following semester. It may also be that dietary goal progress might indicate competence, with individuals reporting greater goal progress feeling more competent, thus leading to more relative autonomous motivation. Indeed, goal progress has been linked to greater satisfaction of basic psychological needs, which is associated with greater autonomous motivation (Milyavskaya et al., 2014).

In the present study, autonomous and controlled motivation were combined into one index measure of relative autonomy. This is a common practice, and in line with self-determination theory’s theoretical view that these forms of motivation are opposite ends of a continuum (Ryan and Deci, 2017). Past research, however, has shown that autonomous and controlled motivation can have different effects on goal pursuit (e.g., Koestner et al., 2008; Milyavskaya et al., 2015; Leduc-Cummings et al., 2022): autonomous motivation is related to more successful goal pursuit and experiencing fewer obstacles. Controlled motivation, on the other hand, is unrelated to goal progress, and setting up and perceiving more obstacles, and greater effort to overcome them. For the present study, we had decided to combine autonomous and controlled motivation into an index of relative autonomy in order to keep model complexity manageable. Perhaps looking at autonomous and controlled motivation separately would have yielded different or more nuanced results. Current investigation examined if introverts and extraverts benefit differentially from specific positive psychology interventions.

### Goal-facilitating behaviours

In examining the effects of goal-facilitating behaviours on goal progress, we found no within-person effects of behaviours on progress. That is, even in those months that individuals used goal-facilitating behaviours, they did not report more progress than their own average. This may be reflective of an inconsistent link between behaviour monitoring and goal progress and attainment more broadly (Harkin et al., 2016). In a meta-analysis examining goal interventions, Harkin et al. (2016) found that monitoring behaviours led to greater behaviour progress, but not necessarily goal progress. In the context of a veg*n diet, this could mean that as a response to engaging in goal-consistent behaviours (e.g., finding vegetarian-friendly restaurants), individuals may dine at vegetarian restaurants more often, but not necessarily maintain a vegetarian diet overall. As such, participants may have perceived themselves to be successful in following their goal diet (e.g., because they dined at a vegetarian restaurant), but may have still eaten animal products on other days and thus did not report objective progress. Additionally, the goal-facilitating behaviours measured in this study were diverse, and included activities such as getting bloodwork done, planning strategies for if a craving occurs, and seeking out veg*n social groups. It may be that engaging in *more* goal-facilitating behaviours than one’s usual was not necessarily better than engaging in *relevant* goal-facilitating behaviours might be.

Additionally, fit between behaviours and a person’s goals might be an important factor in the effectiveness of behaviours for meeting one’s goals. People respond differentially to behavioural interventions based on their personality (Schueller, 2012), and a similar pattern may hold for engaging dietary goal-facilitating behaviours. Outside the context of dietary goals, individuals have been found to favour different strategies, with idiosyncratic personal strategies being more effective than assigned expert strategies at helping people reach their goals (Peetz and Davydenko, 2021). Participants in our study were provided a set of empirically-derived goal-facilitating behaviours, rather than instructed to list personal, idiosyncratic behaviours. It may be that engaging in more goal-facilitating behaviours than one’s own usual is only helpful when these behaviours are consistent with one’s goals or relevant to the individual. Finally, it may be that some people are simply better than others at enacting goal-consistent behaviours (e.g., through implementation intentions) in order to overcome obstacles when pursuing their goal (Koestner et al., 2008).

Indeed, the within-person variance in behaviours was rather low, suggesting that some people regularly enacted more behaviours than others (overall). It may be that people simply do not change their behaviour much month to month, especially if they find something that works. Alternatively, since we assessed number of strategies, it is possible that participants used the same number of strategies month to month, but the strategies themselves differed. When we focused on between-person effects, both in the CLMP model presented in online supplements,^9^ and the correlation between random intercept factors in the RI-CLMP model, the hypothesized relationship between behaviours and progress emerged for the objective measure of goal progress. Together, this suggests that individuals who generally engage in more goal facilitating behaviours tend to make more progress on average compared to others who engage in fewer such behaviours (although they do not perceive this as progress, as indicated by a lack of relation between behaviours and subjective progress).

## Limitations

The measure of goal-facilitating behaviours used in this study has not been previously validated and contains numerous items which measure different constructs (e.g., social support, health monitoring, cost monitoring). Theoretical and empirical research has shown that progress and behaviour monitoring are not monolithic constructs (Wilde and Garvin, 2007; Anseel et al., 2015). Rather, people can assess their behaviour in many ways. Further, behaviour monitoring is most effective at predicting and affecting matching behaviours (e.g., tracking how many times one snacks on cheese may help reduce how often one eats cheese, but may not impact the broader outcome, which is becoming vegan). Future research should better disentangle the various behaviours veg*ns engage in to support their dietary transition. Moreover, certain self-control strategies like implementation intentions are most effective when they are narrow and specific (De Vet et al., 2011). Although participants were asked if they had plans for dealing with cravings, future research would benefit by having individuals transitioning to a veg*n diet make specific, relevant plans for such scenarios.

Additionally, lack of variability with regard to relative autonomous motivation may have influenced the present findings. Participants reported high autonomous motivation and commitment (i.e., intent to continue their veg*n diet after completion of the study; see Anderson and Milyavskaya, 2021; Anderson et al., 2021). New veg*ns in the present study might not represent the general population transitioning to a veg*n diet, who might be more likely to lapse in their diets (Faunalytics, 2016).

In this study, motivation, behaviour, and goal progress were all assessed monthly, and modeled as influencing the following month’s responses. This time scale, however, may not be appropriate (for example, motivation may fluctuate more or less frequently). Using different assessment intervals, and modelling the paths differently, may thus yield different results, although there is currently no reason to expect a different assessment schedule to be more appropriate than what was selected in the current study. Motivations, behaviours, and perceptions are all incredibly fluid and changeable; there is simply not enough research on these fluctuations to properly understand how frequently they may shift and should be assessed.

A final limitation concerns the representativeness of our sample. As described in greater detail in our report on this sample, our participants could be considered representative of those who begin new veg*n diets in Canada and the United States with an initial high level of commitment (Anderson et al., 2021). This means that these findings are not likely to generalize to those who are just experimenting with a veg*n diet, or to individuals in other countries.

## Conclusion

In sum, the present study provides preliminary insights on associations between autonomous motivation, goal-facilitating behaviours, and dietary goal progress in individuals transitioning to a veg*n diet. In line with self-determination theory, autonomous motivation was related with greater progress, but this occurred only when progress was assessed at the same time point; no prospective effects were found. These findings shed light onto the relationship between autonomous motivation, behaviours, and goal progress both at the same time and over time.

## Data availability statement

The datasets presented in this study can be found in online repositories. The names of the repository/repositories and accession number(s) can be found at: https://osf.io/zx4sf/.

## Ethics statement

The studies involving human participants were reviewed and approved by Carleton University Research Ethics Board-B. The patients/participants provided their written informed consent to participate in this study.

## Author contributions

MK and MM came up with the research questions tested in this manuscript. MM and JA designed and conducted the overall larger study. MK conducted the analyses and wrote the manuscript with feedback from MM and JA. All authors contributed to the article and approved the submitted version.

## Funding

This research was supported by Social Sciences and Humanities Research Council of Canada Partnership Engage grant to MM and JA, and a grant from Animal Advocacy Research Fund to Faunalytics.

## Conflict of interest

The authors declare that the research was conducted in the absence of any commercial or financial relationships that could be construed as a potential conflict of interest.

## Publisher’s note

All claims expressed in this article are solely those of the authors and do not necessarily represent those of their affiliated organizations, or those of the publisher, the editors and the reviewers. Any product that may be evaluated in this article, or claim that may be made by its manufacturer, is not guaranteed or endorsed by the publisher.

## Footnotes

^1^We had also preregistered hypotheses on between-person effects, to be tested using cross-lagged panel models (CLMP). However, given the general criticisms of these models (e.g., Lucas, 2022) and the poor fit of these models to our data, on the editor’s recommendation we decided to report the results of these models in an online supplement only: https://osf.io/ez3wx, https://osf.io/bs3qr

^2^Only our variables of interest are reported in this paper. For a full list of variables included in the questionnaires, see https://osf.io/bhksj/

^3^Note that all behaviour categories were combined, as examining each type of behaviour separately was outside the scope of this paper.

^4^Data on subjective and objective progress (and their relation) are used in another manuscript focusing on the correspondence between behavioural and self-report measures of goal progress (Smyth et al., under review).

^5^ https://osf.io/us9bx

^6^ https://osf.io/zx4sf/?view_only=f96dfaabf5ca43b5bc1ec6e1c93eaf96

^7^ https://osf.io/qckva

^8^In the tested models, variance of goal-facilitating behaviours was much higher than the variances of the other variables due to the operationalization in which scores were measured on different scales, resulting in a warning in R. We ran additional analyses with scaled variables. Standardized results remained the same (see online supplement).

^9^ https://osf.io/ez3wx
